# Mitoxantrone-melphalan conditioning regimen for autologous stem cell transplantation in relapsed/refractory lymphoma

**DOI:** 10.3906/sag-1809-36

**Published:** 2019-08-08

**Authors:** Müfide OKAY, Yahya BÜYÜKAŞIK, Haluk DEMİROĞLU, Ümit Yavuz MALKAN, Rafiye ÇİFTÇİLER, Elifcan ALADAĞ, Salih AKSU, İbrahim Celalettin HAZNEDAROĞLU, Nilgün SAYINALP, Osman İlhami ÖZCEBE, Hakan GÖKER

**Affiliations:** 1 Department of Hematology, Faculty of Medicine, Hacettepe University, Ankara Turkey; 2 Department of Hematology, Dışkapı Yıldırım Beyazıt Training and Research Hospital, University of Health Sciences, Ankara Turkey

**Keywords:** Lymphoma, relapsed, autologous stem cell transplantation

## Abstract

**Background/aim:**

High-dose chemotherapy followed by autologous stem cell transplantation (ASCT) has become the standard approach for patients with relapsed/refractory Hodgkin’s lymphoma (HL) or non-Hodgkin’s lymphoma (NHL). In this study, we report the outcome of the mitoxantrone-melphalan conditioning regimen for lymphoma.

**Materials and methods:**

The study group included 53 patients who were relapsed/refractory HL (n = 14) and NHL (n = 39) and received mitoxantrone and melphalan followed by ASCT. The transplant regimen consisted of mitoxantrone (60 mg/m2) and melphalan (180 mg/m2) followed by peripheral blood stem cell infusion (PBSC).

**Results:**

Prior to high-dose chemotherapy, 37.7% of the patients were in complete remission (CR) and 45.3% were in partial remission (PR), and 17% had stable or progressive disease. After high-dose chemotherapy and PBSC, 44 out of 51 patients achieved CR (86.2%). CR was achieved in 24 out of 33 patients (72.7%) who were transplanted in a marginally active phase of the disease. At a median followup of 25.4 months (1.8–131.3 months) after ASCT, 13 patients relapsed/progressed and 8 patients died. The estimated 2-year overall survival (OS) was 81.9%, and event-free survival (EFS) was 59.3%.

**Conclusion:**

High-dose chemotherapy followed by ASCT is an effective conditioning regimen in relapsed/refractory lymphoma patients who are undergoing ASCT.

## 1. Introduction

Although significant advances have been achieved in the treatment of non-Hodgkin’s lymphoma (NHL), 40%–60% of patients still relapse or have treatment-resistant disease [1]. With conventional salvage regimens, these patients have a less than 10% chance of achieving a prolonged disease-free interval. Although the majority of patients with Hodgkin’s lymphoma (HL) will be cured with initial therapy, 5%–10% will not achieve complete remission (CR), and 10%–30% will relapse following standard therapy. High-dose chemotherapy (HDCT) and autologous stem cell transplantation (ASCT) is the most effective treatment and currently the standard approach for patients with relapsed/refractory NHL and HL. Many randomized studies have shown significant improvements in progression-free survival (PFS) and event-free survival (EFS) with ASCT in relapsed/refractory HL and NHL [1–3]. Other regimens, such as BCNU (carmustine), etoposide, cytarabine, melphalan (BEAM), are still more frequently preferred [4–7]. Carmustine, etoposide, cyclophosphamide (CBV), carmustine, etoposide, cytarabine, cyclophosphamide (BEAC), busulfan, cyclophosphamide (BuCy), busulfan, cyclophosphamide, etoposide (BuCyE), total body radiation (TBI) containing regimens (TBI, etoposide, cyclophosphamide), etoposide, and melphalan (VP-16/Mel) are also used in lymphoma [2,5,7–14]. Because of carmustine toxicity, other regimens also used, such as bendamustine containing regimen [bendamustine, etoposide, cytarabine, melphalan (Be-EAM)], fotemustine, etoposide, cytarabine, melphalan (FEAM), thiotepa containing regimen (TEAM), and using mitoxantrone instead of carmustine (NEAM) regimen [14–17]. Carmustine is not a drug that is easy to prescribe due to health policies in many countries, [18] including Turkey. A mitoxantrone-melphalan regimen is preferred in our center instead of BEAM. 

In this retrospective study, we aimed to evaluate the efficacy of mitoxantrone-melphalan as a conditioning regimen before ASCT in patients with relapsed/refractory HL or NHL.

## 2. Methods

### 2.1. Patients 

Fifty-three consecutive adult patients with relapsed/refractory NHL or HL who were treated with mitoxantrone-melphalan and ASCT between June 2001 and December 2017 at Hacettepe University Medical Faculty Hematology Bone Marrow Transplantation Unit have been retrospectively evaluated. All of the ethical considerations had been strictly followed in accordance with the Helsinki declaration. All of the patients provided written informed consent for the ASCT procedure. Patients with primary refractory disease or at least 1 relapse after standard chemotherapy were eligible for the study. Primary refractory disease was defined as disease progression or failing to attain a CR during first-line chemotherapy. Relapsed disease was defined as disease progression after disappearance of all clinical and radiographic evidence of disease for more than 2 months. Relapses occurring within or after 12 months were considered early and late relapse, respectively.

Other eligibility criteria included an Eastern Cooperative Oncology Group (ECOG) performance status of ≤2 and having adequate organ functions defined by a serum creatinine level of <2 mg/dL, serum transaminases less than 2 times the normal value and a bilirubin level of <2 mg/dL, and cardiac ejection fraction greater than 50% determined by echocardiography. Patients had to have adequate pulmonary function, defined as a forced expiratory volume in the first second (FEV 1) and forced vital capacity (FVC) of >80% of the amount predicted. Patients were required to have no central nervous system involvement, and be free of both active infection and human deficiency virus infection. Patients who were not given mitoxantrone-melphalan in the transplant preparation regimen were not included in the study. 

### 2.2. Autologous hematopoietic stem cell transplant regimen

Autologous hematopoietic stem cells were harvested from patients prior to conditioning by either chemomobilization with high-dose filgrastim or high-dose filgrastim alone. Generally we collected 2 × 106 CD34 + cells/kg for the ASCT. The cumulative anthracycline doses were recorded to chemotherapy regimens prior to ASCT. Fifty mg/m2 adriamycin for 1 day for cyclophosphamide, adriamycin, vincristine, prednisone (CHOP); cyclophosphamide, adriamycin, etoposide, vincristine, prednisone (CHOEP); 25 mg/m2 adriamycin for 2 days for adriamycin, bleomycin, vinblastine, dacarbazine (ABVD) regimen, 35 mg/m2 doxorubicin for 1 day bleomycin, etoposide, doxorubicin, cyclophosphamide, vincristine, procarbazine, prednisone (BEACOPP) regimen, and 8 mg/m2 mitoxantrone for fludarabine, cyclophosphamide, mitoxantrone (FCM) were recorded. The mitoxantrone-melphalan regimen consisted of mitoxantrone (given over three 1-h administrations at 1-h intervals on day 4, with a total dose of 60 mg/m2/based on ideal body weight) and melphalan (given over two 1-h administrations at 1-h intervals on day 1, with a total dose of 180 mg/m2/based on ideal body weight) followed by peripheral blood progenitor cell infusion (day 0) [19]. A granulocyte colony stimulating factor was administered at 5 mcg/kg/day subcutaneously beginning on day 2 and continuing until neutrophil recovery.

### 2.3. Supportive care 

All patients received prophylaxis to prevent bacterial, fungal, viral, and pneumocystis carinii infections. Antibacterial and antipneumocystis carinii drugs administered were levofloxacin (500 mg/day, orally) and trimethoprim-sulfamethoxazole (1 double-strength tablet twice a day. 2 days per week), respectively. Ciprofloxacine (500 mg/day) was used as prophylaxis prior to July 2007. Patients received intravenous (IV) fluconazole (400 mg/day) from day +1 until engraftment as an antifungal prophylaxis. The antiviral prophylaxis consisted of IV acyclovir (500 mg/m2/day, in 2 divided doses) from day +1 until engraftment. Irradiated packed red blood cells and single-donor platelet infusions were administered to maintain hematocrit level above 27% and platelet count higher than 20 × 109/L, respectively.

### 2.4. Assessment of response

All patients underwent baseline staging procedures before ASCT, which included physical examination, chest X-ray, computed tomography scans or PET-CT, and bone marrow biopsy. Response to ASCT was determined on posttransplant day +100, and follow-up visits were done every 3 months for the first 2 years and then every 4 months for 3 years. Baseline staging investigations were used as the reference point for subsequent evaluations of disease response. Disease response was defined according to standard lymphoma response criteria [20]. Neutrophil and platelet engraftment were defined as per Center for International Blood and Marrow Transplant Research (CIBMTR) [21].

### 2.5. Statistical analysis

Categorical and continuous variables were expressed as proportions and medians (minimum–maximum), respectively. Survival durations were presented as duration in months ± standard error (SE), 95% confidential interval (CI). The end points of the study were CR rate after mitoxantrone-melphalan and ASCT, overall survival (OS), and EFS. OS was defined as the time from ASCT to the date of death or last follow-up. EFS was defined as at the time from ASCT to treatment failure (no CR or relapse) or death from any cause, whichever occurred first. Disease-free survival (DFS) was calculated in the patients who attained CR from the time of CR to relapse or remission in death. Survival curves were calculated according to the Kaplan–Meier method. A P-value of <0.05 was used as the criterion for statistical significance. SPSS statistics 17 (SPSS Inc., Chicago, IL, USA) was used for statistical analyses.

## 3. Results

### 3.1. Patient characteristics

Clinical characteristics of patients are summarized in Table. The median age of the patients was 45 years (18–68). Of the patients, 73.6% had NHL and 26.4% had HL. The median duration between diagnosis and ASCT was 22.1 months (2.3–88.9). Previous front-line chemotherapy consisted of ABVD and R-CHOP for almost all HL and NHL patients, respectively. Five (9.4%) patients had primary refractory disease, while 41.5% were in early relapse, and 35.8% were in late relapse prior to the salvage regimen (Table). Seven (13.3%) of the patients had cases of lymphoma in its first CR [2 cases of mantle cell lymphoma (MCL); 1 case each of transformed marginal zone lymphoma, Burkitt lymphoma, anaplastic large cell lymphoma, peripheral T cell lymphoma, and diffuse large B-cell lymphoma (DLBCL)]. Salvage chemotherapy was a rituximab (R) when indicated + ifosfamide, carboplatin, and etoposide (ICE) regimen in 33 (64.2%) of patients; dexamethasone, cytarabine, and cisplatin (R-DHAP) in 10 (18.9%) patients; fludarabine, cyclophosphamide, and mitoxantrone (FCM)’ and 1 patient each was treated with BEACOPP, gemcitabine, dexamethasone, and cisplatin (R-GDP), R-CHOP, and sequential R-DHAP/R-Bendamustine. Thirty-two of 53 [18 DLBCL, 5 MCL, 4 follicular lymphoma (FL), 1 Burkitt lymphoma, 1 EBV-associated B-cell lymphoma, 2 T-cell rich B-cell lymphoma, 1 marginal zone to DLBCL] patients with various B cell lymphoma histologies received R-containing salvage regimens. Prior to the mitoxantrone-melphalan regimen, the median cumulative anthracycline dose was 360 mg/m2 (100 mg/m2–505 mg/m2). Six of 39 NHL patients (15.3%) had low intermediate risk while 33 of them (84.7%) had high intermediate risk.

Prior to the transplant regimen, 37.7% and 45.3% of the patients achieved CR and PR after salvage therapy, respectively. Eighty-three percent of patients who were chemosensitive responded to the salvage treatment given before the transplantation. Seventeen percent of the patients underwent transplantation with refractory (7.5%) or progressive (9.5%) disease. The median numbers of mononuclear cells and CD34+ cells infused were 5.6 × 108 (1.1–16.8 × 108) and 5.7 × 106/kg (1.3–42.8 × 106), respectively. Posttransplant median neutrophil and platelet engraftment durations were 11 (9–17) and 14 (7–41) days, respectively. All patients engrafted well.

### 3.2. Response and survival

During a median follow-up of 25.4 months (1.8–131.3) after ASCT, 8 patients died [1 patient due to early transplant-related mortality, 4 patients due to relapse/progressive disease, 3 patients due to other reasons]. At the last follow-up 45 (84.9%) patients were alive. At posttransplant day +100, 44 of 51 evaluable patients were in CR. Two patients in the early posttransplant period were not evaluated. CR ratio was 20/20 (100%), 21/24 (87%), and 3/7 evaluable patients (42%) in patients already in CR, in PR, and having stable/progressive disease at ASCT, respectively (P = 0.001). 

**Figure 1:**
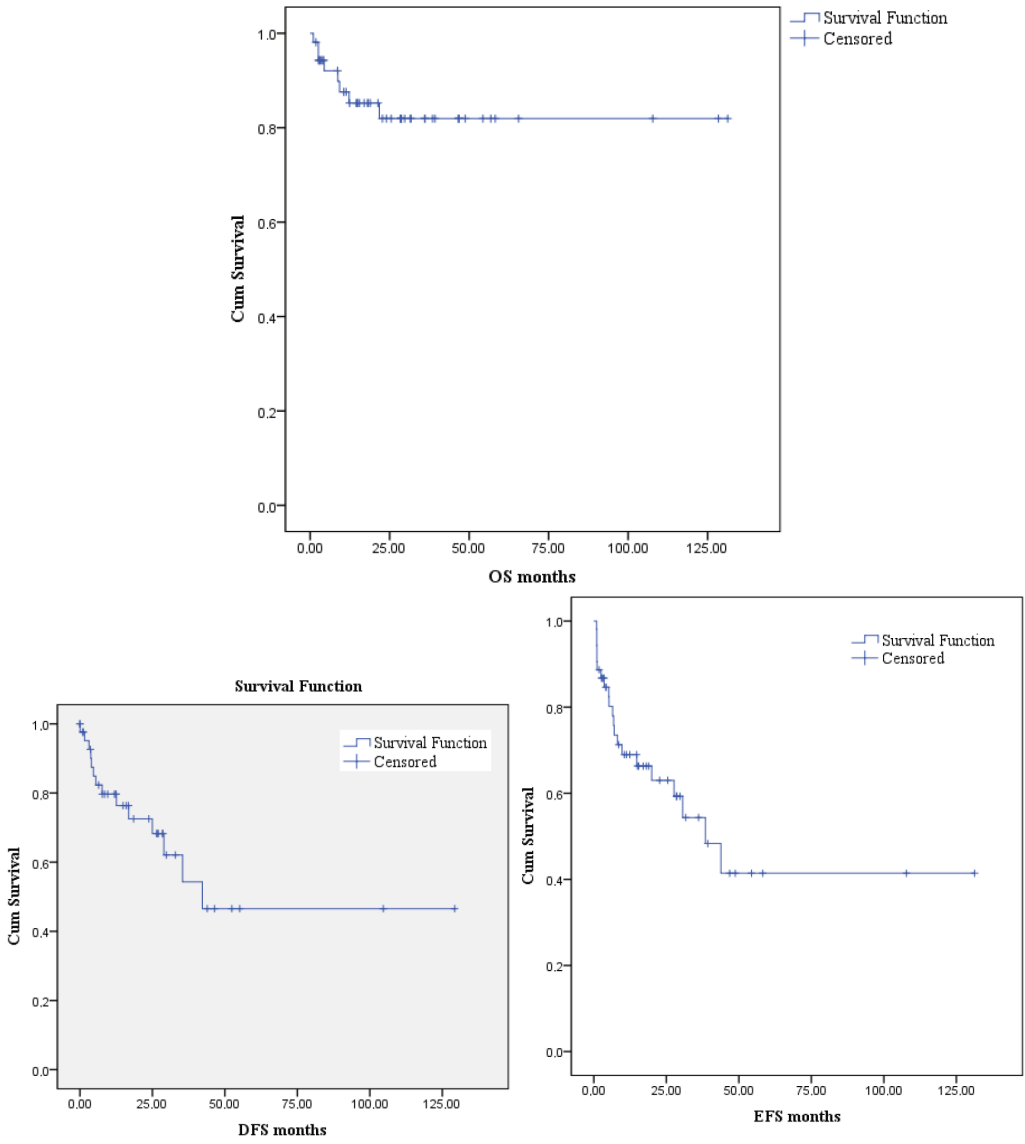
The 5-year OS, DFS, and EFS projections of the patients.

The 5-year OS, DFS, and EFS projections were 81.9%, 46.5%, and 41.4%, respectively. Median OS duration was not reached. Median DFS duration was 42.2 months. Median EFS duration was 38.4 months ± 8.6 (21.5–55.3) (Figure 1). 

**Table:** Characteristics of patients.

	Total (n = 53)
Age	45 (18–68)
Sex MaleFemale	29 (54.7%)24 (45.3%)
DiagnosisHLNHLDLBCLMCLFLT-rich B-cell lymphomaT-cell lymphomaGray zone lymphomaAnaplastic large cell lymphomaBurkitt lymphomaEBV-associated B-cell lymphomaMarginal to DLBCL	14 (26.4%)39 (73.6%)18 (33.9%)6 (11.3%)4 (7.5%)2 (3.7%)3 (5.6%)2 (3.7%)1 (1.9%)1 (1.9%)1 (1.9%)1 (1.9%)
Remission duration prior to salvage (n = 46)*Primary refractoryEarly relapse (≤12 months)Late relapse (>12 months)	5 (9.4%)22 (41.5%)19 (35.8%)
Salvage regimen (n = 46)*R±ICER±DHAPBEACOPPFCMR+GDPR±CHOPR-DHAP/R-Bendamustin	32 (69.5%)9 (19.5%)1 (2.2%)1 (2.2%)1 (2.2%)1 (2.2%)1 (2.2%)
Assessment pre-ASCTCRPRStable (refractory) diseaseProgressive disease	20 (37.7%)24 (45.3%)4 (7.5%)5 (9.5%)

**Figure 2:**
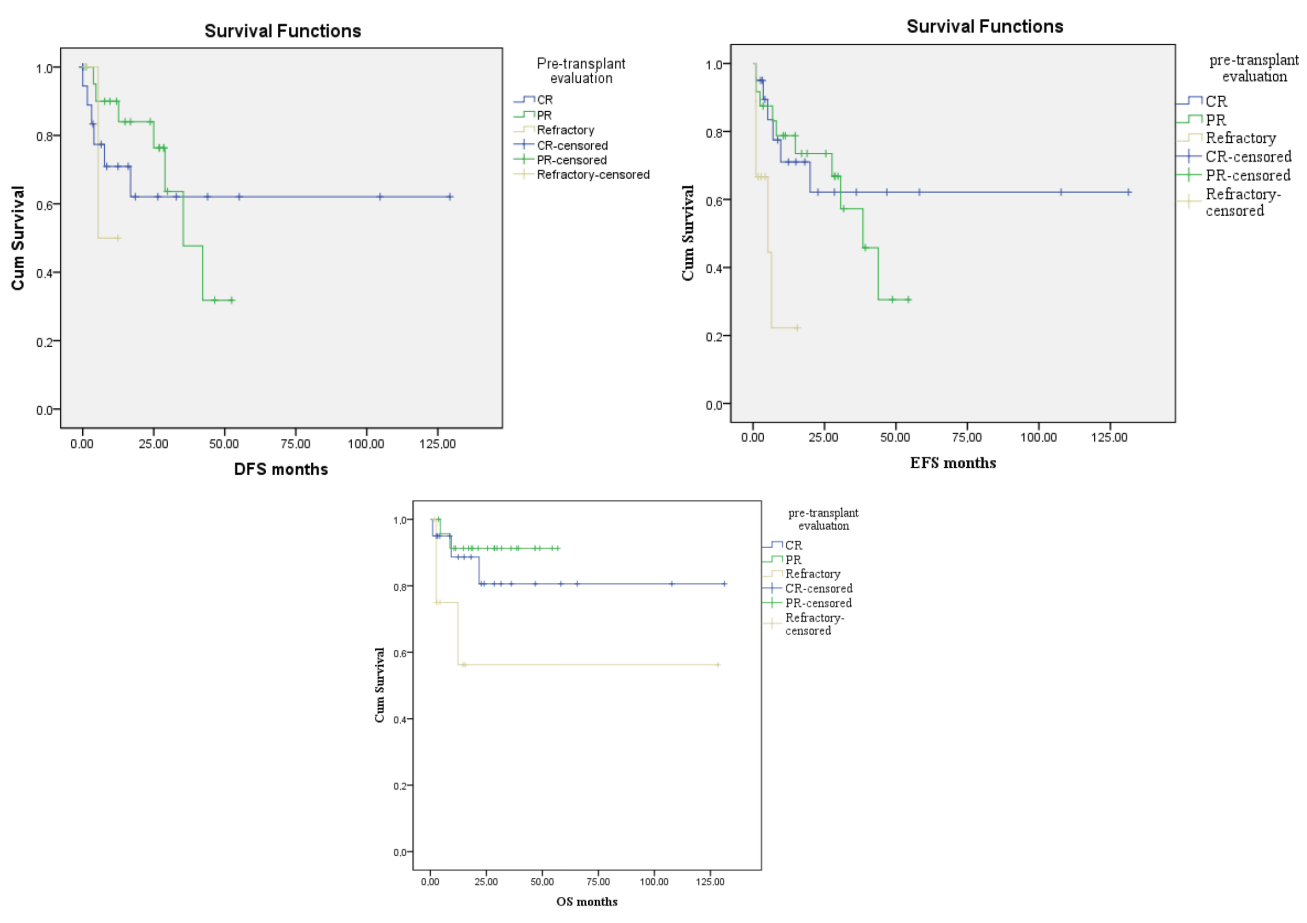
The OS, DFS, EFS durations of the patients according to the pretransplant evaluations.

According to the pretransplant evaluations, OS was 80.6% in the pretransplant CR group, 91.3% in the PR group, and 56.3% in the refractory group. However, the duration of the median OS was not reached in all groups. There was a marginally statistically significant difference between the groups, which showed better OS in patients who are in CR/PR (P = 0.053). DFS was found to be 62.1% in the pretransplant CR group, 31.8% in the PR group, and 50% in the refractory group. Median DFS duration was not reached in the CR and refractory groups and was found to be 35.4 months ± 7.3 (21–49.7) in the PR group. There was no statistically significant difference between the groups (P = 0.67). According to the pretransplantation status, EFS rates were 62.2% in the CR group, 30.5% in the PR group, and 22.2% in the refractory group. In the CR group, median EFS was not reached, in the PR group it was 38.4 months ± 7.1 (24.5–52.4), and in the refractory group it was 5.2 months ± 3.9 (0–12.9). There was a statistically significant difference between the groups (P = 0.01) (Figure 2).

Several factors were evaluated as possible prognostic indicators of OS and EFS. There was no significant survival difference between patients with DLBCL, HL, and others. The 5-year OS and EFS of patients with DLBCL were 75.6% and 39.2%, respectively, which were not significantly different from those of patients with HL (90.9% and 66.7%, respectively) (P = 0.59, P = 0.91).

### 3.3. Toxicity

Major toxicities of the conditioning regimen were grade 3–4 neutropenia and febrile neutropenia. Grade 3–4 thrombocytopenia and neutropenia were observed in all patients. All patients had mucositis, 86% had nausea, and 65% had vomiting. Cardiopulmonary insufficiency was observed in only 1 patient, 29 days after the discharge from the hospital. Since the cumulative dose of anthracyclines that was administered during salvage chemotherapies for the patient was less than 400 mg/m2, the cardiopulmonary insufficiency observed was attributed to a possible viral infection. Seven patients had died 100 days after transplantation. Three of these patients died from sepsis due to pulmonary infection after an average of 6 months, 3 died from disease activation, and the cause of death of 1 patient was not found. One patient underwent an abdominal surgery due to acute intestinal obstruction caused by progressive intraabdominal lymphadenopathy 5 months after the transplantation and died due to an operative complication. The other 2 (3.7%) patients relapsed on day 120 after the acute onset, and died during the 6th month after the onset of neutropenic fever and sepsis with the rescue regimen that started.

## 4. Discussion

Hodgkin’s and non-Hodgkin’s lymphomas are a group of diseases that include B- and T-cell lymphomas and occur in a very wide range of patients. High-dose therapy and ASCT is the standard treatment for relapsed/refractory HL and NHL, based on randomized studies, which showed improved PFS and EFS compared with conventional therapy [1,22–26]. There are also studies showing that in chemosensitive NHL, ASCT is superior to chemotherapy alone in [27,28]. 

In a study comparing the effects of autologous and allogenic transplants on DLBCL, the effect of being chemosensitive and chemoresistant on pretransplantation in DLBCL patients with autologous transplantation was examined. The 4-year OS and PFS rates were 60% and 48%, respectively, and 80% of the patients were chemosensitive. These rates were lower in the chemoresistant group [29]. 

Although our study population was more heterogeneous, our results are comparable with this EBMT study. The CR rate of our cohort who underwent transplantation in marginally active disease (PR/refractory) was found to be 72.7% after ASCT. A median follow-up of 25.4 months was obtained. The 5-year OS and PFS rates were 81.9% and 46.5%, respectively, and 83% of the patients were chemosensitive (CR 37.7%, PR 45.3%). 

In another recent study, the 3-year OS and PFS rates were 86% and 78% respectively, in CR at ASCT HL patients and the other (no-CR) the rates were found to be lower [30]. On the other hand, in our study, the 5-year OS and EFS rates were 90.9% and 66.7%, respectively, in HL patients. However, in our study HL patients were not evaluated before transplantation due to the low number of patients. Similarly, our OS rates in HL were similar to this study although instead we present the overall OS rate, including the chemosensitive and chemoresistant groups together. 

A variety of high-dose conditioning regimens have been available for ASCT in HL and NHL, but no specific regimen has been demonstrated to be superior. Prospective trials have not directly compared to different preparative regimens and settings for ASCT. The choice of conditioning regimen is influenced by patient comorbidities, short- and long-term toxicities, and institutional preferences. In our center’s previous study, a sequential 3-phase regimen in 40 HL and NHL patients has been performed, with conditioning regimen mitoxantrone-melphalan [19]. In that study, the CR rate (35 in 39 evaluable patients) was 90%. The median follow-up was 32 months. The 4-year OS and EFS rates of the patients were 72.2% and 55.3%, respectively [19]. In this study, the OS rate is comparable to the previous study [19]. In another study, the effect of transplant preparation regimen on progression and relapse of NHL and HL autologous stem cell transplant recipients was examined and regimens of HL and NHL were shown to have different effects [5]. The 3-year PFS and OS rates were 51% and 64% in the group using BEAM for NHL. They indicated that BEAM is superior for DLBCL, but not in FL and MCL [5]. On the other hand, in our study, DLBCL group’s 5-year OS rate was 75.6%, and this result is better than BEAM. In patients with HL, the use of Bu/Cy or TBI-based regimens has been shown to increase relapse risk compared to the groups using BEAM or CBV [5]. They have reported the 3-year PFS and OS rates as 62% and 79%, respectively, in HL patients who received the BEAM regimen [5]. In our study, the 5-year EFS and OS rates of HL patients who were given the mitoxantrone-melphalan regimen were superior to the BEAM groups reported in the literature. In [5], the 1-year transplant related mortality (TRM) was found as 4% in patients receiving BEAM, and the TRM was not different in NHL/HL groups with different conditioning regimens. On the other hand, in our study there was only 1 case where possible cardiotoxicity was observed that could be considered a major toxicity. Therefore, in our study the first 100-day mortality rate was found as 1.8%. 

In a recent article, another transplant regimen of lomustine, etoposide, cytarabine, and cyclophosphamide (LACE) was used because BCNU could not be provided. The patient population was heterogeneous [30]. The 3-year OS and PFS were found nearly similar to our present study (70% and 58%). The TRM was found as 7%. 

In another study, bendamustine was used instead of carmustine because it is hard to access carmustine in France. Bendamustine-related severe renal toxicities were observed [31]. In contrast to this, our study demonstrated that mitoxantrone-melphalan could be considered as a somewhat cost-effective regimen for relapsed refractory lymphoma for many reasons. Firstly, no serious toxicity was observed in any of the patients (except only one) with mitoxantrone-melphalan. This data favors the safety of mitoxantrone-melphalan with its low toxicity profile. Secondly, mitoxantrone-melphalan showed favorable OS, EFS, and DFS rates, which confirms the extension of survival durations with the mitoxantrone-melphalan regimen. Thirdly, the regimen should not be a financial disaster for the patients. Mitoxantrone-melphalan is a relatively cheap option. Mitoxantrone is one of the oldest regimens, and it is preferred in our center because it is a cost-effective conditioning regimen option in our country.

To summarize, in our study, 5-year estimated OS and 5-year estimated EFS rates were 81.9% and 41.4%, respectively. The effective use of clinical trials after the relapses after the transplantations could be the reason for the difference between the OS and EFS rates. The OS, EFS, DFS rates according to the response (pretransplant evaluation) given to the salvage chemotherapy regimen before transplantation were compared. The EFS rate was higher in the group with CR before transplantation. The highest OS rate was in the PR group.There was no difference among the groups in rates of DFS. This interesting data could be explained by the tendency of initiation of new generation agents and checkpoint inhibitors (brentuximab vedotin, nivolumab) to PR patients, or faster admission to clinical trials of PR patients compared to CR patients. 

The major limitation of this study is its retrospective design. Another limitation is the low number of patients in both groups. The comparisons regarding the BEAM conditioning regimen could not be performed because of the insufficient number of patients. 

In conclusion, high-dose chemotherapy followed by ASCT is an effective conditioning regimen in relapsed/refractory lymphoma patients who are undergoing ASCT. In this retrospective analysis, the use of the mitoxantrone-melphalan was found to be an effective conditioning regimen for ASCT in relapsed/refractory lymphoma. Future large controlled prospective studies are needed in order to further elucidate the roles of different ASCT conditioning regimen for lymphoma.
